# Mitochondria-Associated MicroRNAs: Emerging Roles in the Pathogenesis of Parkinson’s Disease

**DOI:** 10.3390/biomedicines14020313

**Published:** 2026-01-30

**Authors:** Mariano Catanesi, Luana Di Leandro, Martina Colasante, Annamaria Cimini, Michele D’Angelo, Vanessa Castelli, Cosmin Marian Obreja, Rodolfo Ippoliti

**Affiliations:** 1 Department of Life, Health and Environmental Sciences, University of L’Aquila, 67100 L’Aquila, Italy; luana.dileandro@univaq.it (L.D.L.); martina.colasante@student.univaq.it (M.C.); vanessa.castelli@univaq.it (V.C.); rodolfo.ippoliti@univaq.it (R.I.); 2 Sbarro Institute for Cancer Research and Molecular Medicine, Department of Biology, Temple University, Philadelphia, PA 19122, USA

**Keywords:** MiRNAs, Mito-MiRNA, Parkinson’s disease, mitochondrial dysfunction, agomir, antagomir

## Abstract

Neurodegenerative diseases (NDs) are the most prevalent age-associated disorders, characterized by progressive neuronal loss and cognitive decline. Mitochondrial dysfunction is strictly associated with NDs and represent one of the hallmarks of these disorders, with neurological syndromes frequently representing the primary clinical manifestations of mitochondrial abnormalities. As central regulators of cellular bioenergetics, mitochondria play a pivotal role in both the physiological maintenance and pathogenesis of disease by different regulatory approaches. One of these, microRNAs (miRNAs), a class of small non-coding RNAs, are well-established regulators of gene expression across different biological pathways. These miRNAs were usually investigated within the cytoplasmic context, but recent discoveries have revealed the presence of these miRNAs in different parts of mitochondria, where they contribute to the regulation of gene expression and metabolic activity. These mitochondrial-localized miRNAs, termed mito-MiRNA, may originate from either nuclear or mitochondrial genomes and have been shown to modulate the translational machinery of the cells. Despite extensive research on cytoplasmic miRNAs, the functional roles of mito-MiRNA remain poorly understood, particularly in the context of neurodegenerative disorders. Based on these findings, this review aims to synthesize emerging evidence on the involvement of mito-MiRNA in in one of most prevalent neurodegenerative diseases—Parkinson’s disease (PD).

## 1. Introduction

NDs represent a class of chronic and progressive disorders, characterized by the gradual loss of neuronal function in the central nervous system (CNS). Among these, PD is one of the most prevalent and debilitating pathologies that affect the aging population, and its incidence rate is steadily increasing in parallel with the increase in life expectancy. Clinically, PD manifests through different hallmarks of motor symptoms, such as resting tremors, muscular rigidity, bradykinesia, and postural instability, but structural brain alterations are typically absent in PD patients without dementia. In fact, the onset of PD-associated dementia is accompanied by cerebral atrophy and depigmentation of key brain regions, including the *substantia nigra* and *locus coeruleus*.

Despite extensive investigation, the precise molecular mechanisms driving neurodegeneration remain incompletely understood. One of these mechanisms is characterized by mitochondrial dysfunction that has emerged as a central pathological feature in PD, with evidence indicating that abnormalities in these organelles occur early in disease progression and are nearly ubiquitous among affected individuals.

Aging associated with oxidative stress and impaired mitochondrial bioenergetics are increasingly recognized as critical contributors to neuronal vulnerability, degeneration, and loss of dopaminergic pathway. It can be asserted that mitochondria are indispensable for neuronal survival, regulating adenosine triphosphate (ATP) production, calcium homeostasis, and reactive oxygen species (ROS) generation. The brain’s disproportionately high metabolic demand, consuming nearly ten-fold more oxygen and glucose than other tissues, underscores its reliance on mitochondrial integrity; dopaminergic neurons require an estimated 4.7 billion ATP molecules per second, highlighting their sensitivity to mitochondrial perturbations.

The mitochondrial quality control is maintained through dynamic and finely regulated processes, including biogenesis, mitophagy, fission, and fusion. These processes are orchestrated by a suite of dynamin-related [1] proteins, such as mitofusins (Mfn1 and Mfn2), dynamin-related protein 1 (DRP1), and mitochondrial fission protein 1 (FIS1), which are essential for mitochondrial morphology and stability, genome integrity, and synaptic function. Recent studies have identified short and non-coding RNAs of 19/24 nucleotides as key regulators of mitochondrial dynamics and gene expression. These non-coding RNAs, called mito-MiRNAs, exert regulatory control in the mitochondria, and they require translocation across the organelle’s double membrane to modulate mitochondrial transcriptional activity and other mechanisms. These molecules are gaining attention for their potential as early biomarkers and therapeutic targets in PD, and for these reasons, this review article aims to elucidate the emerging roles of mito-miRNAs in mitochondrial regulation and explore their potential utility as diagnostic biomarkers and therapeutic agents in PD.

## 2. Parkinson’s Disease

The pathological hallmarks of PD include the progressive degeneration of dopaminergic neurons in the *substantia nigra pars compacta*, accompanied by the intracellular accumulation of misfolded alpha-synuclein aggregates, which form Lewy bodies in the cytoplasm of surviving neurons. Clinically, PD is primarily characterized by evident cardinal motor symptoms, such as resting tremors, bradykinesia, muscular rigidity, and postural instability. In addition to these motor signs, a wide spectrum of non-motor symptoms, such as anosmia, hyposmia, depression, cognitive decline, and chronic pain, are increasingly recognized in PD patients.

The pathogenesis of PD is associated to the gradual loss of dopaminergic neurons in the *substantia nigra*, which represents a hub of neural pathways that regulate movements, influencing both voluntary and involuntary action, and neurons present in this area, and is the primary source of brain dopamine. Advancing our understanding of PD necessitates a comprehensive elucidation of its underlying neurodegenerative mechanisms, but the precise origin of neuronal death remains elusive, although current evidence implicates a multifactorial interplay involving genetic predisposition, environmental exposures, and mitochondrial impairment. Notably, mitochondrial dysfunction is increasingly recognized as a central driver in the molecular cascade leading to PD.

A range of mitochondrial dysfunctions [2] have been implicated in the pathophysiology of PD, including the following:

- Excessive generation of ROS, contributing to oxidative damage and cellular stress;
- Disturbance of calcium homeostasis, notably within vulnerable neurons, impairing mitochondrial stress;
- Deficient mitochondrial biogenesis, resulting in reduced mitochondrial renewal and functional capacity;
- Aberrant mitochondrial dynamics, marked by enhanced fission and reduced fusion, which compromise mitochondrial integrity and distribution;
- Impaired mitophagy, hindering the clearance of damaged mitochondria and promoting cellular toxicity;
- Defective axonal transport and mis-localization of mitochondria, disrupting energy supply along neuronal projections and contributing to synaptic dysfunction.

Dopaminergic neurons exhibit exceptionally high metabolic activity and are, therefore, critically dependent on mitochondrial function to sustain their energy requirements. The regulation of mitochondrial homeostasis is modulated by a variety of factors, including mito-MiRNA, which play key roles in post-transcriptional gene regulation, morphology and signaling pathways. Environmental stressors are known to perturb cellular and mitochondrial redox equilibrium: while physiological levels of ROS are essential for intracellular signaling, chronic exposure to elevated ROS concentrations induces oxidative damage to nucleic acids, lipids, and proteins and accelerates mtDNA mutations [3]. High levels of ROS lead to elevated oxidative stress (OS), which is a prominent pathological feature in the PD brain, arising from a loss of capacity of endogenous antioxidant defenses.

Mitochondrial dysfunction further amplifies this process by impairing ATP synthesis and exacerbating ROS generation, thereby establishing a cycle that promotes dopaminergic neurodegeneration.

The clinical diagnosis of PD is primarily based on the presence of hallmark motor symptoms, including bradykinesia, dyskinesia, gait freezing, resting tremor (typically affecting the hands, feet, and jaw), hypomimia, reduced arm swing, and rigidity; while these features are central to diagnosis, complementary diagnostic modalities have gained prominence in recent years. One of these includes neuroimaging techniques, such as magnetic resonance imaging (MRI), transcranial Doppler ultrasonography, positron emission tomography (PET), and single-photon emission computed tomography (SPECT), which play a pivotal role in differential diagnosis [4,5,6,7,8]. Additionally, metabolomic profiling and the identification of neurochemical biomarkers offer promising avenues for enhancing diagnostic accuracy [9,10]. Given the limitations of symptom-based diagnosis, there is an urgent need to develop and validate early diagnostic tools and biomarkers capable of detecting PD in its prodromal stages. In this context, advanced laboratory techniques, such as immunochemiluminescence, real-time quaking-induced conversion (RT-QuIC), and protein misfolding cyclic amplification (PMCA), are emerging as highly sensitive and specific approaches for early and differential diagnosis. Immunochemiluminescence assay [11] is an antibody-based assay coupled with chemiluminescent detection, enabling precise quantification of pathological proteins such as α-synuclein in biological fluids. The RT-QuIC [12] technique detects misfolded α-synuclein by amplifying its seeding activity and monitoring the conversion process in real time, offering exceptional specificity for synucleinopathies. Similarly, PMCA [13] amplifies misfolded protein aggregates through cyclic sonication and incubation, functioning as a “PCR-like” approach for proteins and allowing detection of α-synuclein aggregates even in very small samples. Together, these techniques represent promising tools to improve early diagnosis and guide therapeutic strategies in PD.

Therapeutic management of PD encompasses a multidisciplinary approach, integrating pharmacological, non-pharmacological, and surgical strategies to counteract and slow the progression of disorders. Conventional pharmacotherapy [14] includes agents such as levodopa (often co-administered with carbidopa), dopamine agonists, monoamine oxidase B (MAO-B) inhibitors, catechol-O-methyltransferase (COMT) inhibitors, anticholinergics, and amantadine; all these pharmacological approaches aim to restore dopamine levels. Levodopa remains the cornerstone of PD treatment due to its efficacy in replenishing central dopamine levels. Despite its effectiveness in ameliorating motor symptoms, pharmacological interventions offer limited relief for non-motor manifestations and do not alter disease progression; moreover, long-term use of dopaminergic agents may lead to adverse effects, including motor fluctuations and dyskinesias.

Non-pharmacological therapies serve as essential adjuncts to medical treatment [15] and these include the following: physical therapy (to improve mobility, balance, and strength), occupational therapy (to enhance functional independence), speech therapy, nutritional counselling, and structured exercise programs; such interventions contribute to overall quality of life and functional capacity in PD patients. Finally, surgical intervention [16], particularly deep brain stimulation (DBS), is reserved for patients with advanced disease and refractory motor complications. DBS treatment involves the stereotactic implantation of electrodes into specific brain regions, such as the subthalamic nucleus or *globus pallidus internus*, and these electrodes are connected to an implantable pulse generator located in the thoracic region, which delivers controlled electrical impulses to modulate aberrant neural activity and alleviate motor symptoms.

Emerging therapeutic new strategies, including gene therapy, stem cell transplantation, neuroprotective agents, and nanomedicine [17,18,19,20], represent novel therapeutic opportunities with the potential to modulate disease progression and restore neural function in PD.

## 3. Mitochondrial Dysfunction: Characteristics and Correlation with PD

Mitochondria are multifunctional organelles best known for their role in cellular bioenergetics primarily through the generation of ATP via oxidative phosphorylation and then for several fundamental cellular processes, including calcium signaling, apoptotic regulation, redox homeostasis, and catabolism of carbohydrates and lipids. These organelles exhibit pronounced structural and functional plasticity, continuously undergoing morphological and quantitative changes in response to metabolic demands, and their dynamic nature is governed by tightly regulated processes, such as mitochondrion fusion, fission, and mitophagy, which collectively ensure mitochondrial quality control and adaptability.

Mitochondrial dysfunction is increasingly recognized as a pivotal contributor to the pathogenesis of numerous neurodegenerative disorders; among the clinical phenotypes associated with mitochondrial diseases, neurological and neuromuscular syndromes are particularly prevalent. Despite a relatively lower mitochondrial density in neurons compared to metabolically active cells such as myocytes, the brain exhibits high metabolic demand, consuming oxygen and glucose at rates nearly ten-fold greater than other tissues. Mitochondria are unique among animal cellular organelles that possess their own genome, which encodes 13 essential polypeptides involved in oxidative phosphorylation; however, most mitochondrial proteins are nuclear-encoded and imported post-translationally. Each mitochondrial harbors multiple copies of mtDNA, ranging from 2 to 10 per organelle, resulting in an overall cellular content of approximately 1000 to 100,000 mtDNA copies and, the replication of mtDNA occurs stochastically in heteroplasmic conditions and, selective replication, may favor one variant over another, influencing cellular bioenergetics and disease progression. Protein import into mitochondria is mediated by specialized translocase complexes such as the translocase of the outer membrane (TOM) complex, which identifies and binds mitochondrial targeting sequences, initiating translocation. Subsequently, the translocase of the inner membrane (TIM) complex facilitates the passage of polypeptides into the mitochondrial matrix or their integration into the inner membrane, and these coordinated import mechanisms are critical for maintaining mitochondrial function and integrity, particularly in high-demand tissues such as the central nervous system. The mitochondrial intermembrane space assembly pathway plays a pivotal role in the import and oxidative folding of cysteine-rich proteins within the intermembrane space, and, complementing this, the oxidase assembly machinery mediates the insertion of select proteins synthesized by mitochondrial matrix ribosomes into the inner membrane, thereby contributing to the biogenesis of the respiratory chain complexes.

Mitochondrial dysfunction arises from defects in oxidative phosphorylation [21], typically resulting from mutations in either mtDNA or nuclear DNA; these disorders are broadly categorized as primary mitochondrial diseases, which arise directly from mtDNA mutations, and secondary mitochondrial diseases, which are attributed to impaired nuclear mitochondrial communication leading to mtDNA deletions or depletion. The CNS exhibits increased vulnerability to mitochondrial dysfunction due to several intrinsic factors [22]: first, as previously stated, the brain’s exceptionally high metabolic demand renders it particularly sensitive to deficits in ATP production; second, neuronal tissues possess relatively limited antioxidant capacity, making them more susceptible to oxidative damage induced by ROS; and, third, most neurons are post-mitotic and non-renewable, with neurogenesis restricted to discrete regions, such as the subventricular zone, olfactory epithelium, and hippocampus. These characteristics collectively underscore the critical importance of mitochondrial integrity in maintaining CNS function.

## 4. MiRNAs and Mito-MiRNA: Biogenesis and Trafficking

**Figure 1:**
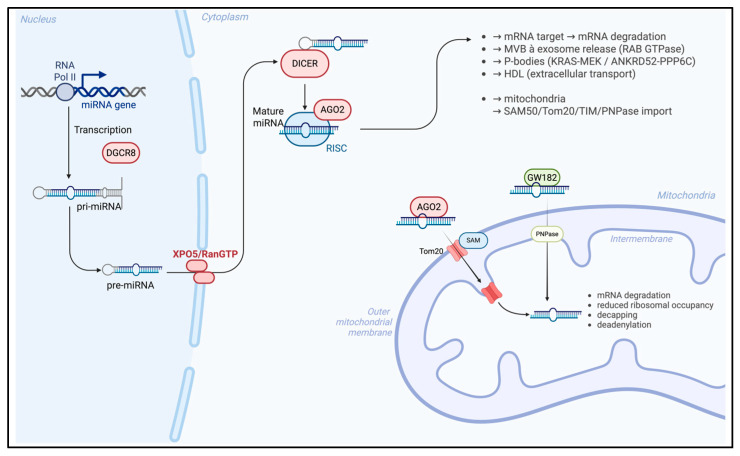
MiRNA biogenesis and trafficking in the nucleus–mitochondria. Created in BioRender. Colasante, M. (2026). https://BioRender.com/918mtnt (accessed on 25 November 2025).

MiRNAs are short non-coding RNA molecules typically ranging from 18 to 25 nucleotides in length, which exert post-transcriptional regulation of gene expression; specifically, miRNAs bind to complementary sequences within target messenger RNAs (mRNAs), leading either to translational repression or to mRNA degradation, depending on the degree of sequence complementarity and the cellular context. Through these mechanisms, miRNAs contribute to the modulation of diverse biological processes, including cell differentiation, proliferation, apoptosis, and stress responses, thereby acting as critical regulators of cellular homeostasis and disease pathogenesis. MiRNAs biogenesis [23] (Figure 1) is a multistep process initiated in the nucleus, where RNA polymerase II and, occasionally, RNA polymerase III, transcribes miRNA genes into primary transcripts (pri-MiRNAs) characterized by a 5′ cap and a 3′ polyadenylated tail; these pri-MiRNAs are processed by the nuclear RNase III enzyme Drosha, in conjunction with its cofactor DGCR8, to generate precursor miRNAs (pre-MiRNAs). After these modification, pre-MiRNAs are subsequently exported to the cytoplasm via the Exportin-5/RanGTP complex, which preserves the structural integrity of the 3′ end during translocation.

In the cytoplasm, the RNase III enzyme Dicer cleaves pre-miRNAs into 21–23 nucleotide duplexes, and one strand of the duplex is selectively incorporated into the RNA-induced silencing complex (RISC), forming the miRNA-RISC complex (miRISC). The role of this complex consists of gene silencing by binding to complementary sequences within the 3′UTR of target mRNA, and Argonaute proteins [24] such as Ago1 and Ago2 are integral components of miRISC and facilitate either mRNA degradation or translational repression. Recent findings [25] have revealed that mature miRNAs can be selectively packaged into extracellular exosomes vesicles, derived from the endosomal pathway, forming exosomal MiRNAs (ex-MiRNAs). The biogenesis of the exosome begins with the formation of early endosomes (EEs) from the plasma membrane, which mature into late endosomes or multivesicular bodies (MVBs) through interactions with the Golgi apparatus, and intraluminal vesicles (ILVs) are formed near the endosomal membrane. MVBs may fuse with lysosomes and can have two different outcomes, as follows: on the one hand, the intraluminal vesicles may be degraded through lysosomal enzymatic activity, thereby contributing to the turnover of cellular components; on the other hand, MVBs can alternatively fuse with the plasma membrane, releasing their intraluminal vesicles into the extracellular space, where they participate in intercellular communication and modulation of the microenvironment. Ras-associated binding (RAB) GTPases are key regulators of MVB trafficking and exosome secretion, thereby influencing the extracellular distribution of ex-miRNAs; this regulatory activity directly impacts the extracellular distribution of ex-miRNAs, thereby modulating intercellular communication, gene expression in recipient cells, and, ultimately, influencing diverse physiological and pathological processes.

Emerging evidence indicates that MiRNAs are not confined to the cytoplasm [26] but are also localized to various intracellular organelles by the sequence motifs near the 3′ end of MiRNAs, which may contribute to their subcellular targeting. In this context, particular attention has been directed toward mito-MiRNA, which are predominantly encoded by the nuclear genome and translocated into mitochondria to modulate the expression of mitochondrial DNA and regulate several fundamental functions.

The precise mechanisms underlying MiRNA import [27] into mitochondria membrane remain incompletely defined; various proteins, such as Ago2, have been implicated and have emerged as a central mediator in this process, being consistently detected within mitochondria across multiple cell types. This protein is thought to contain a mitochondrial targeting sequence near its N-terminal domain, facilitating its translocation into the organelle, and intracellular levels of Ago2 appear to directly influence the efficiency of MiRNA import. Post-translational modifications of Ago2, such as phosphorylation at specific serine residues, may disrupt MiRNA–mRNA interactions and signaling pathways [28], including KRAS-MEK and ANKRD52-PPP6C, and have been implicated in the dissociation of Ago2–MiRNA complexes from RISC, a process mediated via processing bodies (P-bodies), which may contribute to MiRNA trafficking into mitochondria. Several mechanistic models [29] have been proposed to explain MiRNA import into mitochondria; one of these suggests that Ago2–MiRNA complexes cross the outer mitochondrial membrane via SAM50 and Tom20 channels, followed by translocation through the translocase of the inner membrane (TIM) complex.

Another process is driven by GW182 and polynucleotide phosphorylase (PNPase), which may facilitate this process. In this mechanism, PNPase, a 3′ to 5′ exoribonuclease, and poly-A polymerase localized within the mitochondrial intermembrane space play a critical role [30] in regulating MiRNA entry and maintaining mitochondrial homeostasis: once inside the mitochondrial matrix, MiRNAs interact with the 3′UTRs of mitochondrial transcripts [30], modulating gene expression predominantly through silencing mechanisms. These silencing mechanisms include mRNA degradation, reduced ribosomal occupancy, decapping, deadenylation, and interference with cap-binding protein interactions.

Beyond intracellular trafficking, miRNAs are also transported intercellularly by different carry proteins, such as high-density lipoproteins (HDLs), which have been implicated in miRNA delivery near the mitochondrial outer membrane [31]. Short RNA molecules can associate with zwitterionic liposomes, particularly those composed of phosphatidylcholine in the presence of divalent cations such as Ca^2+^ and Mg^2+^ [32]; these interactions can induce conformational changes in lipid headgroups, altering the orientation of aliphatic chains and enabling encapsulation within the lipid bilayer. Although the mechanisms by which HDLs incorporate miRNAs remain to be fully elucidated, it is hypothesized that HDLs may sequester MiRNAs within their hydrophobic core, thereby protecting them from ribonuclease-mediated degradation [33].

Continued investigation is essential to elucidate the molecular contributors of mitoMiRNA biogenesis and trafficking (Figure 1), and a deeper understanding of these processes may facilitate the way for novel therapeutic strategies targeting mitochondrial gene regulation in health and disease.

## 5. Roles of Mito-MiRNAs in Parkinson’s Disease

This section explores the key biological pathways through which mito-MiRNA may modulate the pathophysiological progression of PD. The transcriptomic profiling [34] has revealed approximately 125 microRNAs exhibiting differential expression in the prefrontal cortex of PD patients, as reported by Hoss et al., indicating a complex regulatory network potentially governed by miRNAs in neurodegenerative processes [34].

Among the central mechanisms implicated in PD pathogenesis are oxidative stress, which impairs antioxidant defenses; mitochondrial quality control, which dysregulates dynamics mitophagy; and neuroinflammation, which amplifies neuronal injury. For these reasons, this chapter focuses on the principal mito-MiRNA associated with these three interrelated processes, elucidating their roles in the initiation and advancement of PD (Table 1).

### 5.1. Role of Mito-MiRNA in Modulating Oxidative Stress

**Figure 2:**
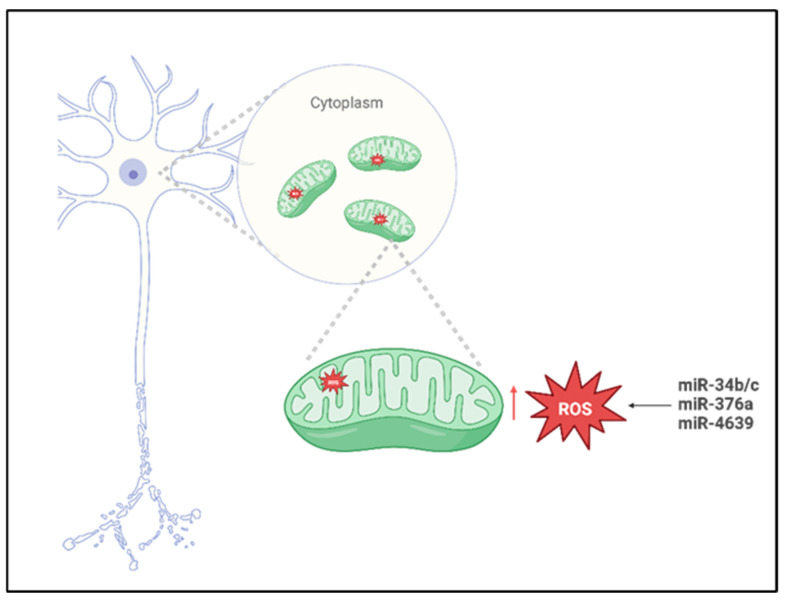
Mito-MiRNA associated with mitochondria ROS imbalance in Parkinson’s disease. Created in BioRender. Colasante, M. (2026). https://BioRender.com/918mtnt (accessed on 25 November 2025).

In a study conducted by Miñones-Moyano et al. [35], postmortem brain tissue sampled from the amygdala and frontal cortex were analyzed using reverse transcription polymerase chain reaction (RT-PCR). The cohort analyzed in this study included individuals with advanced-stage PD, early-stage PD, and neurologically healthy controls. The primary objective for the researchers was to quantify the expression levels of miR-34b/c across these groups, and the analysis revealed a significant downregulation of miR-34b/c in both PD groups compared to the controls. To further investigate the functional implications of this dysregulation, the authors performed different in vitro experiments using a neuroblastoma cell line, such as SHSY-5Y, transfected to suppress the expression of miR-34b/c. The results obtained demonstrated that reduced levels of miR-34b/c compromised the cellular capacity to mitigate and scavenging ROS accumulation (Figure 2), indicating a critical role in oxidative stress regulation. Based on these findings, the authors proposed that miR-34b/c may directly target *Parkin*, *DJ-1*, and *PTEN-induced kinase 1* (*PINK1*), three genes critically involved in mitochondrial quality control and PD pathogenesis, thereby linking miRNA dysregulation to neurodegenerative mechanisms underlying the disease.

In a study conducted by Baghi et al., [36], venous blood samples were obtained from individuals stratified into three clinical groups—neurologically healthy controls, patients with early-stage PD, and patients with advanced-stage PD—classified according to their degree of motor disability. In this research, quantitative analyses were performed to assess both ROS levels and the expression of miR-376a; the results obtained revealed a significant upregulation of miR-376a in both PD cohorts relative to healthy controls, accompanied by markedly elevated ROS production. Notably, increased miR-376a expression correlated with the downregulation of genes critical for mitochondrial homeostasis, including *peroxisome proliferator-activated receptor gamma coactivator 1-alpha* (*PGC1-α*) and *glycogen synthase kinase 3 beta* (*GSK3-β*). These findings suggest that miR-376a may play a pivotal role in modulating mitochondrial integrity and oxidative stress responses and that its dysregulation appears to contribute to the pathological accumulation of ROS (Figure 2), thereby promoting oxidative-stress-induced neuronal damage and cell death in PD.

Cheng et al. [37] conducted a comprehensive investigation employing miRNA microarray profiling followed by validation in plasma samples from a cohort comprising 169 sporadic PD patients, 170 neurologically healthy controls, and 60 individuals diagnosed with essential tremor. The analysis obtained from MiRNA profiling, revealed a significant increase in miR-4639-5p levels in PD patients, and this miRNA demonstrated influence in identifying early-stage PD cases from individuals with advanced-stage PD from healthy individuals. Subsequent mechanistic studies [49] indicated that miR-4639-5p exerts post-transcriptional repression of *DJ-1*, a gene implicated in PD pathogenesis and known for its neuroprotective role against oxidative stress. In familial PD, the L166P loss-of-function mutation in *DJ-1* compromises protein [50] stability, impairs its antioxidative function, and reduced DJ-1 protein levels; this case has also been documented in sporadic PD cases [51], although the underlying regulatory mechanisms remain incompletely understood. Elevated expression of miR-4639-5p [52] was found to correlate with decreased DJ-1 protein levels, leading to increased oxidative stress (Figure 2) and neuronal vulnerability. Based on these findings, miR-4639-5p shows promise as a peripheral biomarker for early PD diagnosis and a potential molecular target for therapeutic intervention aimed at restoring redox homeostasis and neuroprotection. The selective passage of molecules through the inner and outer mitochondrial membranes represents an important mitochondrial parameter implicated in increased OS and neuronal degeneration [53].

The mitochondrial permeability transition pore (mPTP), a multiprotein complex located on the mitochondrial membrane, plays a pivotal role in stress-induced cell death such as ROS, by facilitating the release of pro-apoptotic factors. In this context, MiR-7, a small non-coding RNA, has demonstrated neuroprotective properties in experimental models of PD; recent findings indicate that miR-7 modulates mitochondrial membrane permeability from the external side [38,54] by downregulating *voltage-dependent anion channel 1* (*VDAC1*), a key structural component of the mPTP. To confirm these results, the researchers performed experimental overexpression of miR-7 in a neuroblastoma cell line and murine neurons subjected to oxidative stress, resulting in a significant reduction in mitochondrial damage, intracellular calcium release, and ROS accumulation. Functional silencing of *VDAC1* reiterated the protective effects of miR-7, whereas reintroduction of *VDAC1* lacking its miR-7-responsive regulatory sequence abrogated these advances. These observations suggest that miR-7 exerts its neuroprotective effects by directly targeting *VDAC1*, thereby preserving mitochondrial integrity and attenuating stress-induced apoptotic signaling.

### 5.2. Mito-MiRNA Implicate in Mitochondria Quality Control

**Figure 3:**
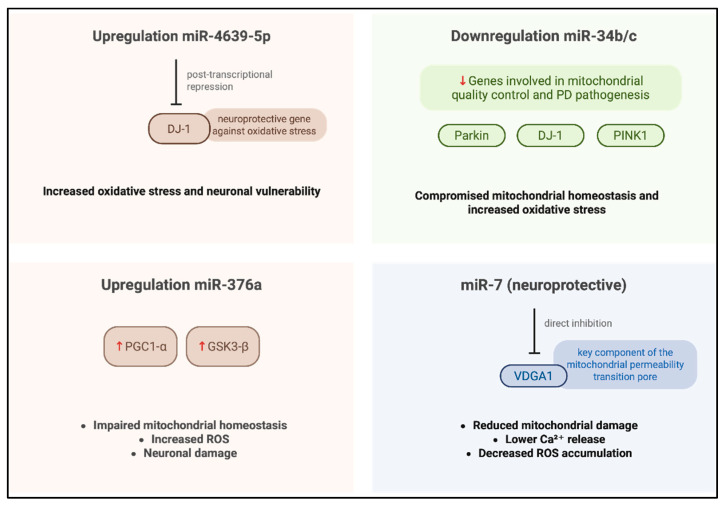
Regulation of mito-MiRNA in mitochondrial homeostasis. Created in BioRender. Colasante, M. (2026). https://BioRender.com/918mtnt (accessed on 25 November 2025).

An additional critical pathway in which mito-MiRNA are implicated is mitophagy, the selective autophagic removal of damaged mitochondria [55], which is essential for preserving mitochondrial integrity and sustaining cellular homeostasis (Figure 3); indeed, the clearance of dysfunctional mitochondria is vital for maintaining mitochondrial quality control and ensuring efficient energy production. Disruptions in mitophagy and mitochondrial apoptosis are known to exacerbate the degeneration of dopaminergic neurons [56], which are particularly dependent on intact mitochondrial function for survival and synaptic activity.

MiRNAs called miR-27a and miR-27b have been implicated in the regulation of mitophagy through their direct interaction, at the level of the outer membrane, with *PINK1* [39], a serine/threonine kinase essential for the selective autophagic clearance of damaged mitochondria. Kim et al. demonstrated that miR-27a/b suppresses human *PINK1* expression via translational inhibition, targeting the 3′UTR of its mRNA [39]; this repression attenuates lysosome-mediated mitochondrial clearance, thereby modulating mitophagy flux. Under physiological conditions, miR-27a/b contribute to the maintenance of mitochondrial integrity by keeping PINK1 protein levels low, thus preventing unwarranted mitophagy. In response to mitochondrial damage, these miRNAs act as modulators of the mitophagy pathway, fine-tuning PINK1 expression and serving as molecular gatekeepers; notably, their expression is upregulated under conditions of sustained mitophagy stress, suggesting a compensatory feedback mechanism aimed at limiting excessive mitochondrial degradation. Elucidating the regulatory dynamics of miR-27a/b may provide critical insights into *PINK1*-dependent mitophagy and its broader implications for mitochondrial quality control and cellular homeostasis.

Another important miRNA that has been shown to be implicated in the regulation of mitophagy is MiR-21, which modulates the expression of *PINK1*. In fact, this miRNA has been identified as a negative regulator of mitophagy, acting through modulation of the PINK1/Parkin signaling axis. Under mitophagy-inducing damage ions, PINK1 phosphorylates both ubiquitin and the E3 ubiquitin ligase Parkin, initiating the recruitment of autophagic machinery to damaged mitochondria; conversely, phosphatase and tensin homolog (PTEN) antagonizes this pathway by dephosphorylating ubiquitin [57], thereby disrupting the downstream signaling cascade required for mitophagic progression [41].

A notable contribution to these studies was made by Cheng et al., [58] who investigated the role of miR-181a in regulating mitophagy and mitochondrial apoptosis. In their work, overexpression of miR-181a in a neuroblastoma cellular model led to a marked suppression of mitophagic activity, evidenced by impaired degradation of damaged mitochondrial proteins. Furthermore, elevated levels of miR-181a increased cellular susceptibility to apoptosis induced by mitochondrial uncoupling agents; conversely, inhibition of miR-181a conferred protection against cell death. These findings suggest that miR-181a modulates mitochondrial quality control mechanisms and may contribute to neuronal vulnerability under conditions of mitochondrial stress, highlighting its potential relevance in neurodegenerative processes such as PD.

Di Rita et al. [42] investigated the role of miR-218 in the regulation of mitophagy and identified *PRKN* (Parkin RBR E3 ubiquitin ligase) as a novel direct target. *PRKN* is a critical component of mitochondrial quality control and has been extensively implicated in the pathogenesis of various neurodegenerative disorders, including PD. Dysregulation of PRKN activity has been linked to miR-218, which directly targets PRKN, adding a new layer of post-transcriptional regulation to mitochondrial homeostasis, suggesting that aberrant miRNA expression may exacerbate PD progression by disrupting mitophagy pathways. To confirm this, functional assays [42] revealed that MiR-218 downregulates *PRKN* mRNA expression in HEK293 cells, leading to a reduction in mitochondrial ubiquitylation, an essential step in the autophagic clearance of damaged mitochondria, and this disruption impairs mitophagic flux and compromises mitochondrial turnover [59].

Beyond mitophagy, mitochondrial-derived vesicles (MDVs) represent an additional mechanism for maintaining mitochondrial homeostasis [60]. MDVs are formed through the selective packaging of mitochondrial components into vesicles that are subsequently trafficked to lysosomes for degradation and the lysosomal delivery of MDVs is dependent on the PINK1/PRKN [61] axis, further underscoring the centrality of this pathway in mitochondrial quality control. Given the shared regulatory mechanisms between mitophagy and MDV trafficking, miR-218 emerges as a candidate for modulating mitochondrial surveillance systems [62]. Its ability to target PRKN defines it as a potential regulator of both mitophagic activity and vesicle-mediated mitochondrial turnover, with implications for therapeutic intervention in PD and related neurodegenerative conditions.

An in vitro study by Xiong et al. [43] have demonstrated that overexpression of miR-494 significantly reduces DJ-1 protein levels, thereby increasing cellular vulnerability to oxidative stress. To validated these findings, Xiong et al. [43] used an MPTP-induced mouse model of PD [43], where elevated miR-494 expression was associated with downregulation of *DJ-1* and exacerbation of neurodegenerative outcomes. These observations suggest that miR-494-mediated suppression of *DJ-1* may contribute to impaired antioxidant defense mechanisms, promoting dopaminergic neuronal loss. The pathological regulation of *DJ-1* by miRNAs such as miR-494 may, therefore, represent a critical molecular event in the cause of sporadic PD, highlighting the potential of miRNA-targeted strategies for therapeutic intervention.

Liu et al. [40] investigated the neuroprotective role of miR-124 in dopaminergic neurons using both in vivo and in vitro PD, including MPTP-treated mice and SH-SY5Y cells exposed to MPP^+^. To enhance miR-124 expression, the researchers delivered complementary oligonucleotides for enhancement of miR-124; this therapeutic intervention effectively mitigated dopaminergic neuronal loss and maintained striatal dopamine concentrations in MPTP-treated mice. From a mechanistic point of view, the study [40] identified Bcl-2-interacting mediator of cell death (Bim) as a direct target of miR-124, implicating it in the regulation of apoptotic signaling. The treatment with complementary oligonucleotides for enhancement miR-124, suppressed Bim expression, thereby inhibiting Bax translocation to the mitochondria, a key event in the intrinsic apoptotic pathway. Furthermore, miR-124 overexpression ameliorated autophagic dysfunction, as evidenced by decreased autophagosome accumulation and restoration of lysosomal integrity in both experimental models.

Collectively, these findings demonstrate that miR-124 exerts neuroprotective effects by modulating apoptosis and autophagy, highlighting its therapeutic potential in reducing dopaminergic neurodegeneration associated with PD.

### 5.3. Mito-MiRNA Involved in the Regulation of Inflammatory Processes

**Figure 4:**
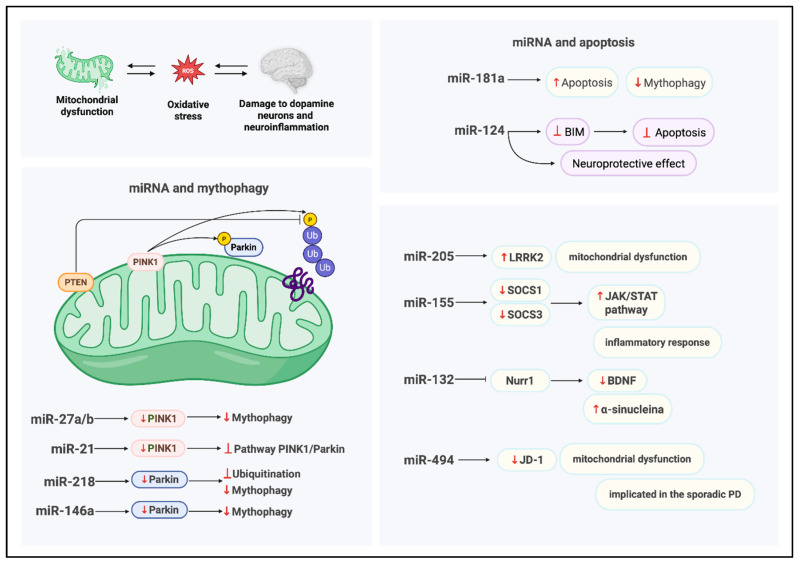
Functional targets and roles of mito-MiRNA in Parkinson’s disease. Created in BioRender. Colasante, M. (2026). https://BioRender.com/918mtnt (accessed on 25 November 2025).

*Leucine-rich repeat kinase 2* (*LRRK2*) has emerged as a critical molecular player in elucidating the pathogenesis of PD. This is a multifunctional protein implicated in several fundamental cellular processes, including cytoskeletal dynamics, intracellular vesicle trafficking, lysosomal integrity, mitochondrial homeostasis, and immune regulation (Figure 4). Pathogenic mutations in the *LRRK2* gene are a well-established cause of autosomal dominant PD.

Recent findings by Cho et al. [63] demonstrate that microRNA-205 (miR-205) directly targets *LRRK2* via a conserved binding site located at the 3′-UTR of its mRNA. They first analyzed brain tissue from Parkinson’s disease patients and controls, finding that LRRK2 protein levels were elevated in PD samples, while miR-205 expression was significantly reduced, suggesting an inverse relationship. Using luciferase reporter assays, they confirmed that miR-205 binds to a conserved site in the 3′-UTR of *LRRK2* mRNA, repressing its translation. Further in vitro experiments showed that overexpression of miR-205 in HEK293T cells and primary neurons markedly decreased LRRK2 protein levels. Importantly, restoring miR-205 in neurons expressing pathogenic *LRRK2* mutants rescued neurite outgrowth deficits, indicating a functional role in mitigating PD-related cellular damage. These findings establish miR-205 as a key post-transcriptional regulator of *LRRK2* and suggest its potential as a therapeutic target in Parkinson’s disease. Notably, individuals with sporadic PD exhibit significantly reduced miR-205 expression accompanied by elevated LRRK2 protein levels, suggesting a dysregulation of this post-transcriptional control mechanism.

Further in vitro studies [64] using primary neuronal cultures and cell lines corroborate the regulatory role of miR-205 in modulating *LRRK2* expression, underscoring its potential relevance in PD pathophysiology. In a study conducted by Caggiu et al. [45], the expression profile of miR-155 was investigated in patients with PD, revealing a marked upregulation associated with inflammatory processes. To assess its functional role, they performed inhibition experiments in animal models using complementary oligonucleotides targeting miR-155. This intervention reduced neuroinflammation and alleviated disease symptoms, demonstrating that miR-155 contributes to PD pathogenesis and highlighting its potential as a therapeutic target. MiR-155 exerts a pro-inflammatory effect by downregulating key anti-inflammatory regulators, including suppressor of cytokine signaling 1 and 3 (SOCS-1 and SOCS-3); these molecules regulate inflammation by inhibiting JAK/STAT signaling pathways, which are crucial for immune responses. Experimental evidence from animal models [65] indicates that targeted inhibition of miR-155 using complementary oligonucleotides can attenuate disease symptoms, underscoring its therapeutic potential.

Notably, Caggiu et al. [45] were the first to report elevated miR-155 levels in peripheral blood mononuclear cells of PD patients, a finding that agrees with prior observations in α-synuclein-based PD mouse model. These data suggest that miR-155 plays a critical role in orchestrating the neuroinflammatory response to α-synuclein aggregation. Furthermore, accumulating evidence positions miR-155 as a key regulator of neuroinflammation, particularly in response to α-synuclein oligomers. Supporting this, miR-155 knockout mice exhibit significantly attenuated inflammatory responses following exposure to α-synuclein fibrils [66], reinforcing its involvement in PD pathogenesis and highlighting its potential as a molecular target for therapeutic intervention.

Lungu et al. [46] analyzed miRNA expression in a spontaneous autosomal recessive rat model of neurodegeneration and found miR-132 to be significantly upregulated in the mesencephalon. This finding was validated through qRT-PCR. Subsequent analyses focused on miR-132′s downstream target, the transcription factor Nurr1, which is essential for midbrain dopaminergic neuron development. Western blotting and immunohistochemistry revealed decreased Nurr1 protein levels in affected rats. Additionally, BDNF, regulated by Nurr1, was significantly reduced in both mesencephalic tissue and serum. These results suggest that elevated miR-132 suppresses *Nurr1* and *BDNF* expression, potentially contributing to dopaminergic neuron dysfunction and neurodegeneration. This decline was attributed to the combined effects of α-synuclein overexpression and impaired Nurr1-mediated transcriptional activity, suggesting a mechanistic link between miR-132 dysregulation and neurotrophic support deficits in PD-like neurodegeneration.

Jauhari et al. [47] used several experimental approaches in their study. First, they performed global miRNA profiling on brain tissue from a rotenone-induced Parkinson’s disease rat model to identify differentially expressed miRNAs associated with inflammation. They then validated the upregulation of miR-146a using qRT-PCR. To explore the mechanism, they assessed NF-κB activation and phosphorylation through Western blotting, followed by chromatin immunoprecipitation (ChIP) to confirm NF-κB binding to the miR-146a promoter region. Finally, they examined the functional consequences of miR-146a induction on inflammatory signaling pathways, linking oxidative stress to miRNA-mediated regulation of neuroinflammation. This research, in Ref. [47], identified miR-146a as the most significantly upregulated miRNA in response to neuroinflammatory stimuli; elevated levels of miR-146a were shown to suppress mitophagy by downregulating *Parkin*, a key E3 ubiquitin ligase involved in mitochondrial quality control. The resulting decrease in Parkin expression impairs the clearance of damaged mitochondria, leading to their accumulation and increased production of ROS, which, in turn, contributes to neuronal injury and degeneration.

These findings underscore the pivotal role of miR-146a in linking neuroinflammation to mitochondrial dysfunction in PD pathogenesis.

### 5.4. Transfer RNA Fragments

In addition to canonical microRNAs, recent research [67] has uncovered other classes of small regulatory RNAs that exhibit miRNA-like functions. Among these, tRNA-derived fragments (tRFs) have attracted considerable attention. These molecules originate from the cleavage of mature tRNAs or their precursors and were recently recognized as a distinct and functionally active family of small non-coding RNAs. tRFs are classified based on [68] their cleavage sites and origin within tRNAs and include different subtypes such as tRF-5, tRF-3, tRF-2, tRF-1, and i-tRF. Similar to miRNAs, tRFs interact with Ago proteins [69] to regulate gene expression post-transcriptionally. Through this association, they influence mRNA stability and translation by guiding the RISC complex to repress or degrade target transcripts. While some tRFs particularly 3′ tRFs bind strongly to Ago proteins, others, such as 5′ tRFs, exhibit weaker or distinct Ago preferences. This interaction underpins the miRNA-like functions of tRFs, affecting protein synthesis and cellular stress responses, although loading efficiency and regulatory mechanisms vary across tRFs subtypes and cellular contexts. Evidence suggests that tRFs [70] are involved in diverse cellular processes, including stress responses, metabolic regulation, and inter-organelle communication. Regarding PD, the role of tRFs is poorly understood but several works have initiated investigations into their functional roles and behavioral patterns across various NDs. Zhang et al. [71] demonstrated that tRFs are downregulated in Alzheimer’s disease mouse (AD) models, contributing to mitochondrial dysfunction, a key feature of both AD and PD. These fragments influence oxidative phosphorylation and mitochondrial translation by targeting mitochondrial mRNAs or ribosomal proteins. Oxidative stress, induced by factors such as heavy metals or H_2_O_2_, promotes cleavage of mature tRNAs, increasing tRF production. These fragments can either support stress adaptation or contribute to cell death. For example, tRF-Glu-CTC is modulated under oxidative stress and influences neurogenesis [72], whereas 5′ tRF-Gly-GCC can exacerbate ROS generation. Thus, oxidative stress not only drives tRNA cleavage and dynamic tRF regulation, balancing translation and cellular homeostasis, but can also be amplified by tRF activity, creating a feedback loop that accelerates disease progression.

## 6. MicroRNAs as Emerging Biomarkers for Parkinson’s Disease

Recent advances [73] in molecular diagnostics have highlighted miRNAs as promising candidates for early detection of PD; their remarkable stability in extracellular environments, coupled with disease-specific expression patterns, underscores their potential utility in biomarker development. Circulating miRNAs, including miR-193b [74], miR-150 [75], miR-124-3p [76], and miR-4639-5p [44] actively secreted by cells and detectable in various biofluids, such as cerebrospinal fluid, plasma, serum, saliva, urine, and pleural fluid, offer a minimal invasive approach for monitoring disease onset and progression. This paradigm shifts toward pre-symptomatic diagnosis parallels established strategies in other chronic diseases, such as diabetes mellitus [77], where biomarkers like fasting blood glucose enable stratification into healthy, pre-diabetic, and diabetic states. Analogously, miRNA profiling in PD could inform clinical decision-making, support risk stratification, and enable personalized therapeutic interventions. Although preventive treatments for PD remain limited, miRNA-based diagnostics hold considerable promise for improving early detection, mitigating symptom progression, and enhancing patient outcomes.

Based on current evidence, the following mito-MiRNA are considered leading candidates for the diagnosis of Parkinson’s disease:

- miR-144-5p [48]: demonstrates consistent upregulation in the cerebrospinal fluid of PD patients across all stages of disease progression, implicating its involvement in α-synuclein aggregation;
- miR-27a-3p [78]: exhibits reduced expression in early-stage PD, suggesting its potential utility as an early diagnostic marker;
- miR-145-3p [79]: identified in saliva, where its overexpression contributes to DJ-1 suppression and oxidative stress, indicating its supplementary diagnostic relevance;
- miR-214 [80]: shows elevated levels during the prodromal phase of PD, with a subsequent decline in advanced stages, reflecting a negative correlation with disease severity;
- miR-485-3p [80]: significantly upregulated in the serum of PD patients relative to individuals with Alzheimer’s disease and healthy controls, underscoring its diagnostic specificity;
- miR-4639-5p [44]: increased in plasma and derived from central nervous system exosomes; its expression is associated with oxidative stress through *DJ-1* downregulation.

Additional miRNAs, including miR-7 [81], miR-103-3p [82], miR-153 [83], and miR-124 [84], have also demonstrated dysregulated expression profiles in PD, further supporting their potential as circulating biomarkers for disease monitoring and patient stratification.

## 7. MicroRNAs as Potential Therapeutic Agents

In addition to their emerging role as diagnostic biomarkers, miRNAs are increasingly recognized for their hypothetical therapeutic potential in neurodegenerative [73] disorders, particularly PD; their ability to modulate entire molecular networks and gene expression, makes them attractive candidates for therapeutic where multiple pathogenic pathways, such as mitochondrial dysfunction, oxidative stress, neuroinflammation, and α-synuclein aggregation, converge.

In PD, dysregulated miRNA expression has been implicated in the disruption of neuronal homeostasis, dopaminergic neuron survival, and synaptic function. Restoring or inhibiting specific miRNAs (Table 2) can, therefore, rebalance these pathways and potentially halt or reverse disease progression. The miRNA modulators under investigation, known as agomirs and antagomirs, are still in the early stages of development, with research currently limited to in vitro and in vivo models [85].

- Agomirs (miRNA mimics) [86]: These are chemically engineered oligonucleotides designed to mimic the function of endogenous miRNAs that are downregulated in PD. By reintroducing functional miRNA molecules, agomirs can restore normal gene regulation. For example, mimicking miR-7 miRNA known to suppress α-synuclein expression may reduce toxic protein aggregation in dopaminergic neurons.
- Antagomirs (miRNA inhibitors) [87]: These are antisense oligonucleotides that bind to and inhibit overexpressed miRNAs contributing to PD pathology. For instance, inhibition of miR-4639-5p, which downregulates *DJ-1* and promotes oxidative stress, could alleviate neurodegenerative processes by restoring antioxidant defenses.

Key therapeutic agomirs candidates include the following:

- miR-214 agomir [80]: reduces α-synuclein aggregation and oxidative stress, showing neuroprotective effects in both in vitro and in vivo PD models;
- miR-144-3p agomir [88]: enhances mitochondrial biogenesis by targeting APP and upregulating PGC-1α;
- miR-7 agomir [89]: mitigates mitochondrial fragmentation and inflammation by modulating mPTP and NLRP3 inflammasome activity;
- miR-124 agomir [90]: promotes autophagy and cell survival by downregulating pro-apoptotic Bim.

While therapeutic antagomirs candidates include the following:

- miR-146a antagomir [91]: which plays a key role in regulating immune responses, inflammation, and angiogenesis by enhancing NF-κB pathway;
- miR-103a-3p antagomir [90]: by binding to the 3′-UTR of Parkin mRNA, miR-103a-3p reduces *Parkin* and *Ambra1* expression, impairing mitochondrial clearance and contributing to neurodegeneration;
- miR-181a/b antagomir [90]: inhibits the p38 MAPK/JNK signaling pathway, reducing cell death and autophagy markers like LC3II and Beclin-1;
- miR-494-3p antagomir [90]: restores *SIRT3* expression, leading to improved mitochondrial function, reduced oxidative stress and inflammation, enhanced neuronal survival, and better motor performance in Parkinson’s disease models.

**Table 2:** **Effects of Agomirs and Antagomirs on Molecular Targets and Cellular Functions**.

Agomir	Taget	Function	References
miR-214 agomir	Synuclein	Reduce synuclein aggregation	[80]
miR-144-3p agomir	PGC-1α	Enhances mitochondrial biogenesis	[88]
miR-7 agomir	mPTP, NLRP3	Mitigates mitochondrial fragmentation and inflammation	[89]
miR-124 agomir	Bim	Promotes autophagy	[90]
**Antagomir**	**Taget**	**Function**	**References**
miR-146a antagomir	NF-κB	Regulating immune responses, inflammation, and angiogenesis	[91]
miR-103a-3p antagomir	Parkin	Impairing mitochondrial clearance	[90]
miR-181a/b antagomir	MAPK/JNK	Reduced cell death and autophagy	[90]
miR-494-3p antagomir	SIRT3	Reduced oxidative stress and inflammation	[90]

Despite their promise, miRNA-based therapies face several challenges, including delivery across the blood–brain barrier, off-target effects, and immune activation. Recent advances in nanoparticle-based delivery systems, viral vectors, and exosome-mediated transport have shown potential in overcoming these barriers. Targeted delivery to specific brain regions and cell types remains a critical area of ongoing research.

## 8. Discussion

Recent studies have increasingly highlighted the role of mito-miRNAs in the pathophysiology of PD. These small non-coding RNAs, localized inside mitochondria, exhibit a unique dual regulatory capacity modulating gene expression in both nuclear and mitochondrial genomes. This influence allows mito-miRNAs to orchestrate key processes, such as mitochondrial biogenesis, oxidative phosphorylation, and mitophagy, all of which are critically impaired in PD.

Despite the identification of over 400 miRNAs within mitochondria [92], several mechanistic aspects remain unresolved. These include the pathways governing the import of nuclear-encoded miRNAs into mitochondria, the specific molecular targets of mito-miRNAs within mitochondrial transcripts and the extent to which individual mito-miRNAs contribute to disease-specific mitochondrial dysfunction. Furthermore, it is unclear whether mito-miRNAs act independently or synergistically to regulate mitochondrial activity [59] across different neurodegenerative conditions, including Alzheimer’s disease (AD), Huntington’s disease (HD), and PD.

Addressing these gaps is essential for advancing our understanding of mitochondrial gene regulation and for identifying novel therapeutic strategies aimed at restoring mitochondrial integrity. Given the central role of mitochondrial dysfunction in PD, mito-miRNAs may serve not only as biomarkers for disease progression but also as direct targets for therapeutic intervention.

## 9. Conclusions and Future Perspectives

The expanding field of mito-MiRNA research is shedding new light on the molecular mechanisms underlying PD. Mito-miRNAs, a distinct subset of miRNAs localized within mitochondria, have demonstrated the capacity to regulate translational processes across both nuclear and mitochondrial genomes. This dual-site regulatory function underscores their critical role in preserving mitochondrial homeostasis and cellular energy balance. Their integration into key signaling pathways, such as those governing mitochondrial biogenesis, oxidative phosphorylation, and mitophagy positions.

As evidence accumulates, mito-miRNAs are increasingly recognized as powerful tools for dissecting the complex molecular interplay between mitochondrial dynamics and neurodegeneration. Despite these advances, the precise mechanisms by which mito-miRNAs influence gene expression and contribute to PD pathophysiology remain incompletely understood.

Continued investigation into mito-miRNAs gene interactions is essential not only for refining our understanding of PD but also for guiding the development of targeted disease-modifying therapies. Their unique regulatory properties offer a promising line of research for therapeutic innovation in PD and other neurodegenerative disorders.

## Figures and Tables

**Table 1:** Details of mito-MiRNA implicated in Parkinson’s disease.

Mito-MiRNA	Expression Regulation	Target	Function	References
miR-34b/c	Downregulation	Parkin, DJ-1, PTEN	Decrease ROS scavenging	[35]
miR-376a	Upregulation	PGCQ-A, GSK3-b	Increase ROS production	[36]
miR-4639-5p	Upregulation	DJ-1	Increase ROS production	[37]
miR-7	Downregulation	VDAC1	Increase mithocondrial dysfunction	[38]
miR-27a/b	Downregulation	PINK-1	Mitophagy regulation	[39]
miR-21	Upregulation	PINK-1, Parkin, PTEN	Mitophagy regulation	[40]
miR-181a/b	Upregulation	PINK-1	Mitophagy regulation	[41]
miR-218	Upregulation	PRKN	Mitophagy regulation, vesicles trafficking	[42]
miR-494	Upregulation	DJ-1	Increase ROS vulnerability	[43]
miR-124	Upregulation	Bcl-2, Bax	Regulation of apoptotic signalling	[40]
miR-205	Upregulation	LRRK2	Mitochondrial homeostasis	[44]
miR-155	Upregulation	SOCS-1/3, JAK/STAT	Increase inflammation	[45]
miR-132	Upregulation	Nurr1, Synucelin	Synuclein accumulation	[46]
miR-146a	Upregulation	NF-kB	Increase inflammation	[47]
miR-144-5p	Upregulation	NF-kB	Increase inflammation	[48]
miR-145-3p	Upregulation	DJ-1	Increase ROS production	[37]
miR-214	Downregulation	KLF4, Synuclein	Synuclein accumulation	[46]
miR-103-3p	Downregulation	Parkin, Ambra1	Mitochondrial homeostasis	[44]

## Data Availability

No new data were created or analyzed in this study.

## Abbreviations

The following abbreviations are used in this manuscript:

NDs	Neurodegenerative diseases
miRNAs	MicroRNAs
mito-MiRNAs	Mitochondrial-localized miRNAs
PD	Parkinson’s disease
CNS	Central nervous system
ROS	Reactive oxygen species
ATP	Adenosine triphosphate
Mfn	Mitofusins
Drp1	Dynamin-related protein 1
Fis1	Mitochondrial fission protein 1
mtDNA	Mitochondrial DNA
MRI	Magnetic resonance imaging
PET	Positron emission tomography
SPECT	Single-photon emission computed tomography
MAO-B	Monoamine oxidase B
COMT	Catechol-O-methyltransferase
DBS	Deep brain stimulation
TOM	Translocase of the outer membrane
TIM	Translocase of the inner membrane
pri-MiRNAs	Primary transcripts
pre-MiRNAs	Precursor miRNAs
RISC	RNA-induced silencing complex
miRISC	miRNA-RISC complex
Ago1/Ago2	Argonaute proteins
ex-MiRNAs	Exosomal MiRNAs
EEs	Early endosomes
ILVs	Intraluminal vesicles
PNPase	Polynucleotide phosphorylase
HDLs	High-density lipoproteins
LRRK2	Leucine-rich repeat kinase 2
Nurr1	Nuclear receptor-related 1 protein
